# Structural mechanisms of chaperone mediated protein disaggregation

**DOI:** 10.3389/fmolb.2014.00012

**Published:** 2014-09-15

**Authors:** Rui Sousa

**Affiliations:** Department of Biochemistry, University of Texas Health Science Center at San AntonioSan Antonio, TX, USA

**Keywords:** protein aggregation, chaperones, heat shock proteins, Hsp70, Hsp110, ClpB, Hsp104, AAA+ proteins

## Abstract

The ClpB/Hsp104 and Hsp70 classes of molecular chaperones use ATP hydrolysis to dissociate protein aggregates and complexes, and to move proteins through membranes. ClpB/Hsp104 are members of the AAA+ family of proteins which form ring-shaped hexamers. Loops lining the pore in the ring engage substrate proteins as extended polypeptides. Interdomain rotations and conformational changes in these loops coupled to ATP hydrolysis unfold and pull proteins through the pore. This provides a mechanism that progressively disrupts local secondary and tertiary structure in substrates, allowing these chaperones to dissociate stable aggregates such as β-sheet rich prions or coiled coil SNARE complexes. While the ClpB/Hsp104 mechanism appears to embody a true power-stroke in which an ATP powered conformational change in one protein is directly coupled to movement or structural change in another, the mechanism of force generation by Hsp70s is distinct and less well understood. Both active power-stroke and purely passive mechanisms in which Hsp70 captures spontaneous fluctuations in a substrate have been proposed, while a third proposed mechanism—entropic pulling—may be able to generate forces larger than seen in ATP-driven molecular motors without the conformational coupling required for a power-stroke. The disaggregase activity of these chaperones is required for thermotolerance, but unrestrained protein complex/aggregate dissociation is potentially detrimental. Disaggregating chaperones are strongly auto-repressed, and are regulated by co-chaperones which recruit them to protein substrates and activate the disaggregases via mechanisms involving either sequential transfer of substrate from one chaperone to another and/or simultaneous interaction of substrate with multiple chaperones. By effectively subjecting substrates to multiple levels of selection by multiple chaperones, this may insure that these potent disaggregases are only activated in the appropriate context.

## Introduction

Molecular chaperones carry out a wide variety of cellular protein processing reactions, but are most familiar for their roles in preventing protein aggregation. Indeed, their roles in inhibiting protein aggregation remind us that their designation as chaperones emerged from the recognition that they are involved in “preventing *inappropriate* interactions,” and that the first protein so designated was nucleoplasmin (Laskey et al., 1993), which assists in forming proper nucleosome (DNA:histone) complexes by inhibiting formation of incorrect ones. The latter observation highlights the fact that chaperones are involved not only in inhibiting general, heterogeneous protein aggregation, but also in inhibiting the formation of specific misassembles and, by doing so, facilitate the formation of functional assemblies.

**Figure 1:**
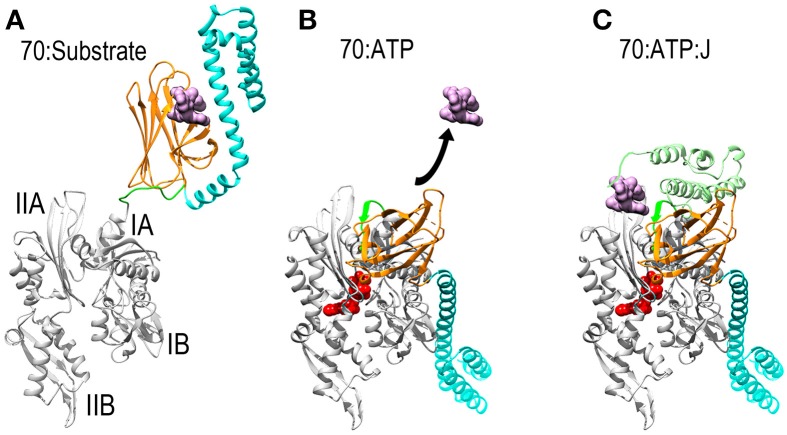
**Mechanisms of ATP dependent protein substrate release and binding by Hsp70. (A)** Ribbon model of Hsp70 ADP state structure [pbd 2KHO (Bertelsen et al., 2009)] with the NBD in gray, PBD-β and PBD-α in orange and cyan, respectively, and the linker between the NBD and PBD in green. A space-filling model of substrate peptide (magenta) is shown bound to the PBD. **(B)** Model of Hsp70 ATP state structure [pdb 4B9Q (Kityk et al., 2012)]. Binding of ATP induces NBD to close around the nucleotide which expands the groove between subdomains IA and IIA, allowing the interdomain linker to bind in this groove. This creates a binding site for PBD-β, which separates from PBD-α and releases the protein substrate. **(C)**: Hsp70:ATP:J domain structure [modeled from pdb 2QWQ (Jiang et al., 2007)]. The J domain of a J protein (pale green) binds to the IIA-Linker-IA surface induced by ATP binding and its distinctive substrate binding domain binds and present a protein substrate to Hsp70. Upon ATP hydrolysis the J protein releases Hsp70 which clamps down on the protein substrate to return to the conformation shown in **(A)**.

The mechanism by which a chaperone can inhibit inappropriate protein:protein interactions is most easily seen in the chaperonins. These proteins assemble into large, hollow cylinders that enclose a space into which an unfolded protein can be admitted (Langer et al., 1992). Thus isolated, the enclosed protein is free to fold but is prevented from aggregating with other proteins. Cycles of ATP hydrolysis by the chaperonin control recruitment and release of proteins from the folding chamber (Martin et al., 1993). The two other major mechanisms by which chaperones inhibit protein aggregation involve the non-ATP hydrolyzing HSPs and the ATP-hydrolyzing chaperones of the Hsp70 family [most chaperones are also designated heat shock proteins because, upon application of heat shock or other cellular stresses, their expression increases to handle the increased amounts of unfolded and aggregating proteins that accumulate under such conditions (Welch, 1987)]. The non-ATP hydrolyzing HSPs display hydrophobic surfaces or pockets which can bind and shield exposed hydrophobic segments of mis- or unfolded proteins, thus preventing them from aggregating until they can fold to sequester these hydrophobic segments and be released from the chaperone (Lundin et al., 2004). The Hsp70s contain a trap-like protein binding domain (PDB) which opens and closes in response to ATP binding and hydrolysis in their nucleotide-binding domains (NBD; Figure 1) (Bertelsen et al., 2009; Kityk et al., 2012). Depending on its degree of closure, the PBD can bind extended hydrophobic polypeptide segments or small misfolded protein domains with exposed hydrophobic regions (Marcinowski et al., 2011). When bound to Hsp70, misfolded proteins are shielded from aggregating with other proteins and, upon their ATP-driven release from the chaperone, are free to fold into their native states.

All these mechanisms, in one way or another, sequester exposed hydrophobic patches on a protein to inhibit its aggregation. Most of the early mechanistic and structural studies on chaperones focused on understanding how these sequestration mechanisms work. The possibility that chaperones might also function by disaggregating and refolding proteins *after* they had clumped together was not as great a focus. To a certain degree this was for technical reasons: while recent work has revealed that certain classes of chaperones can dissociate aggregates, their ability to do so is affected by the physical parameters of the aggregates such as their size and degree of compaction and secondary structure. Neither the appreciation of this fact nor controlled methods for preparing different types of aggregates were present during the earlier period of work on chaperones, nor was it understood that effective disaggregation usually involves cooperation between different types of chaperones and co-chaperones (while sequestration and inhibition of aggregation can often be effected by a single chaperone). In addition, it would have been easy to accept the argument that protein disaggregation, unlike inhibition of aggregation, would be a mechanistically improbable thing for a protein to do, both in terms of energetics (aggregates would be too stable to be readily dissociated) and mechanics (how does one protein grab another and pull it out of an aggregate?).

However, about 15 years ago, work began to appear reporting that chaperones could disaggregate and refold proteins, and more recent work is revealing the mechanisms of these surprisingly potent disaggregation reactions. This review focuses on what we know about the structural mechanisms of protein disaggregating chaperones. These include the Hsp104/ClpB molecules, which are representative of the AAA+ class of proteins, and the Hsp70/Hsp110 chaperones. Both of these two chaperone types use ATP hydrolysis to drive the mechanical work of protein disaggregation, but they do so by different mechanisms. For each class of chaperone, reactions in which the chaperones act on heterogeneous protein aggregates, and reactions in which they dissociate well-defined and functionally specific protein complexes will be described in an effort to reveal the common mechanistic features of these seemingly disparate reactions. The mechanical process of protein disaggregation will also be compared to another mechanical action mediated by chaperones—the translocation of unfolded proteins through pores and into (and out of) the ER and mitochondrion—to see if such a comparison will illuminate the mechanisms of these processes which would otherwise seem similar only in that they are executed by the same type of protein. Finally, the recent discovery of a small non-ATP hydrolyzing Hsp that can break apart a defined protein aggregate, and therefore presents an exception to the paradigm of ATP-dependent disaggregases, will be described.

## The Hsp70 chaperones and protein disaggregation

### Mechanism of Hsp70 protein substrate binding and release

Thanks to a number of recent NMR and X-ray structures, the mechanism by which Hsp70 binds and releases protein substrates is now understood. Hsp70 is composed of a horseshoe shaped NBD (Flaherty et al., 1990), and a PBD composed of β-sandwich pocket and α-helical lid subdomains (Zhu et al., 1996) (Figure 1A). In its nucleotide-free or ADP states the NBD assumes an open and flexible conformation. ATP binding induces the NBD to close around the nucleotide (Woo et al., 2009). This closure involves a hinging motion between subdomains IA and IIA and, just as the hinging motion of a door alters the space between the door and the jamb, NBD closure expands the space between subdomains IA and IIA and alters the disposition of the surface of these two domains relative to each other (Bertelsen et al., 2009; Bhattacharya et al., 2009). The consequence of this is to create a binding site for the β-sandwich pocket of the PBD (PBD-β) and the highly conserved, hydrophobic linker that connects the NBD and PBD. This linker is exposed and protease sensitive in the absence of ATP (Jiang et al., 2005; Vogel et al., 2006a), but the hinging motion between subdomains IA and IIA expands the space between these domains, allowing the linker to bind in the groove between them as part of an extended β-sheet. The PBD-β subdomain then binds to the surface created by subdomains IA, IIA and the interdomain linker that sits between them. The docking of PBD-β onto this surface displaces PBD-α, and the helical lid subdomain settles onto the subdomain IB of the NBD (Figure 1B). Since PBD-α acts as a lid that keeps proteins bound to PBD-β from dissociating, the separation of these two subdomains—coupled to conformational changes induced in PBD-β by gain of interactions with the NBD and/or loss of interactions with PBD-α—opens the PBD to allow bound proteins to be released. The PBD is then available to bind another protein substrate, which is usually presented to it by a J protein, a protein characterized by the presence of a J domain and a distinct domain that binds different protein substrates (Szabo et al., 1994; Misselwitz et al., 1998). The J domain binds to the Hsp70 IA-linker-IIA-PBD-β surface that is allosterically induced by ATP binding (Jiang et al., 2007; Ahmad et al., 2011; Kityk et al., 2012). Via mechanisms that are still not fully understood, but which exemplify the remarkably tight coordination of reaction steps in chaperone mediated processes, binding of the J protein and its associated protein substrate to the Hsp70 synergistically stimulate ATP hydrolysis (Szabo et al., 1994; Vogel et al., 2006b; Kityk et al., 2012). Hydrolysis of ATP allows the NBD to open, closing the groove between subdomains IA and IIA to displace the linker and PBD-β. This, in turns, allows the PBD-α lid to associate with PBD-β, effectively clamping down on the bound protein substrate to block its dissociation. There appears to be significant flexibility in the degree of closure of the helical lid which allows Hsp70 to bind both fully unfolded polypeptide segments and denatured globular protein domains or molten globule states (Marcinowski et al., 2011; Schlecht et al., 2011).

In the ADP state, interactions between the PBD and NBD are dynamic with these domains both transiently associating with each other as well as assuming dissociated conformations in which the two domains do not interact and remain connected only by the interdomain linker (Bertelsen et al., 2009; Mapa et al., 2010; Marcinowski et al., 2011). The protein substrate:Hsp70 complex with ADP bound is relatively stable with its lifetime limited by the rate at which ADP dissociation allows subsequent ATP binding (Ha and McKay, 1995; Takeda and McKay, 1996; Brehmer et al., 2001). Nucleotide exchange factors (NEFs) bind and open the NBD to allow ADP release, following which ATP can bind to drive Hsp70 through further cycles of protein release and binding (Rampelt et al., 2011).

### Hsp70 mediated protein disaggregation

Hsp70 mediated protein disaggregation has been studied most extensively with the bacterial proteins DnaK (Hsp70), DnaJ (the bacterial Hsp40 J protein), and GrpE (the bacterial NEF) (Schroder et al., 1993; Diamant et al., 2000; Ben-Zvi et al., 2004). The disaggregation action of this combination of chaperones is not especially vigorous and its effectiveness varies greatly with different protein substrates and different methods of preparation of the protein aggregates. The most salient features of DnaK/DnaJ/GrpE mediated disaggregation are (Ben-Zvi et al., 2004): (1) A stoichiometric excess (~5-fold) of DnaK over substrate to achieve maximal disaggregation and refolding efficiency is required. This has reasonably been interpreted in terms of a need for multiple Hsp70 molecules binding to a single unfolded protein to cover its interactive regions and inhibit its re-aggregation to allow its refolding after it has been extracted from the aggregate. If just the disaggregation step in the reaction is examined then maximal efficiency is achieved at a ~1:1 DnaK to substrate ratio. It should be appreciated, however, that this still represents an abnormally large stoichiometric ratio for an enzymatic reaction, and that under these conditions DnaK is in large excess of aggregate particles. (2) DnaJ is essential in the reaction, with optimal efficiency occurring with DnaJ at 10–20-fold less than DnaK, indicating that DnaJ is essential for recruiting DnaK to the aggregate but acts catalytically with a single DnaJ loading multiple DnaK molecules onto the aggregate. (3) GrpE is stimulatory, but not essential, for the disaggregation reaction and also acts catalytically with optimal DnaK:GrpE ratios in the range of 20-fold.

#### Hsp110, a potent Hsp70 family disaggregase

**Figure 2:**
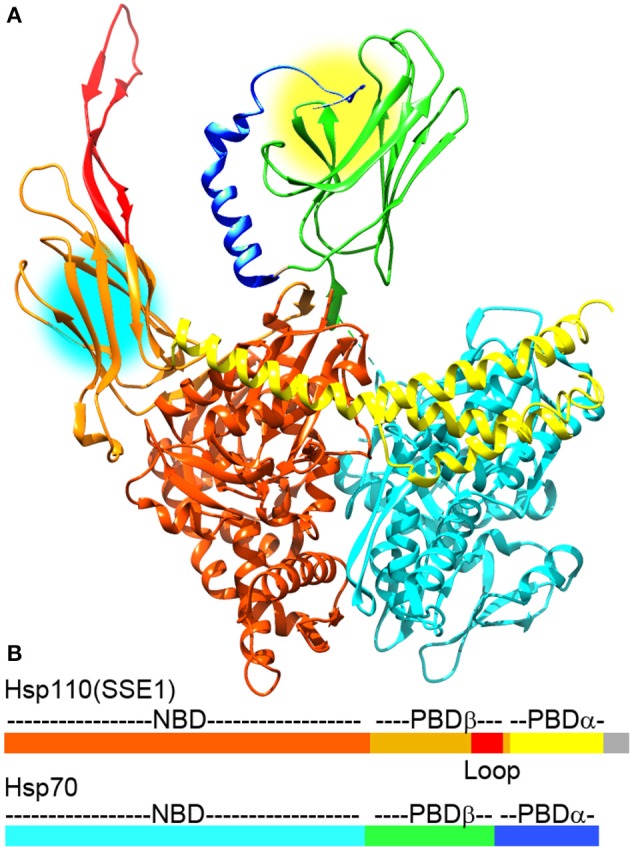
**The Hsp110:Hsp70 complex may cooperate in protein unfolding and disaggregation. (A)** Ribbon model of the complex between Hsp110 and Hsp70 [pdb 3C7N (Scheurmann et al., 2008)]. The NBD, PBD-β, acidic insertion loop and PBD-α of Hsp110 are colored brown, orange, red and yellow, respectively. The NBD, PDB-β and PBD-α of Hsp70 are in light cyan, green, and blue, respectively. Though Hsp110 acts as a NEF for Hsp70 in this complex, both Hsp110 and Hsp70 PBDs can bind substrate proteins and are near enough in the complex (protein binding sites are highlighted by yellow and cyan circles) that they could bind a single substrate or pass a substrate between them to enhance their protein unfolding and disaggregation activities. **(B)** Domain structure of Hsp110 and Hsp70 with coloring corresponding to the ribbon models (gray area in Hsp110 corresponds to a disordered C-terminal region that is not visible in the X-ray structures).

Though disaggregation reactions mediated by Hsp70 are relatively inefficient, it has recently been discovered that the combination of Hsp70 and Hsp110 acts as a much more effective disaggregase (Shorter, 2011; Rampelt et al., 2012). Hsp110 (Sse1 and 2 in yeast) was first identified as an exceptionally divergent Hsp70 family member (sequence identity between Hsp70s is typically ~45%, but is less than ~30% between Hsp110 and Hsp70) (Mukai et al., 1993). The largest single difference between Hsp70 and Hsp110 is the insertion of a negatively charged ~100 residue sequence (the acidic insertion loop) between strands 7 and 8 of the β-sandwich subdomain of the Hsp110 PBD (Figure 2). Hsp110 did not initially appear to go through the same cycles of ATP regulated protein substrate binding and release as Hsp70, nor did it act as genuine unfolding/refolding ATPase. It was shown, however, that it could act as an effective “holdase,” binding and holding denatured proteins so as to inhibit their aggregation (Shaner et al., 2004; Hrizo et al., 2007). Hsp110 was also found to act as NEF for Hsp70, a discovery which led to a focus on its role as the most abundant and important of the Hsp70 NEFs in metazoans (Shaner et al., 2005, 2006; Dragovic et al., 2006; Raviol et al., 2006; Andreasson et al., 2008; Morgan et al., 2013). However, contrary to initial reports that Hsp110 does not undergo ATP regulated cycles of protein substrate binding/release, it has recently been found that yeast Hsp110 (Sse1) affinity for peptide substrates is reduced by ATP to a similar degree as Hsp70, indicating that ATP regulates yeast Hsp110 and Hsp70 protein substrate binding kinetics in similar ways (Xu et al., 2012). The major differences in substrate binding properties between yeast Hsp110 and Hsp70 are that the kinetics of binding are much faster with Hsp110, and that Hsp110 binds preferentially to peptides rich in aromatic residues, while Hsp70 prefers aliphatic rich sequences. But, despite the fact that yeast Hsp110 substrate binding activity is regulated by ATP similarly to Hsp70, yeast Hsp110 cannot refold denatured luciferase in the same way as Hsp70 can, but is effective at inhibiting denatured luciferase aggregation (Xu et al., 2012).

In contrast, mammalian Hsp110 has recently been shown to function similarly to Hsp70 in that it can refold and recover the activity of denatured proteins in an ATP and Hsp40 dependent fashion (Mattoo et al., 2013). It is unclear if or how the differences in substrate binding kinetics and sequence preferences of Hsp70, yeast or mammalian Hsp110 are responsible for their differential abilities in refolding denatured proteins, but the greater potency of mammalian vs. yeast Hsp110 in this respect may correlate with the differences in these proteins' disaggregase activities. Mammalian Hsp110, together with mammalian Hsp70 and Hsp40, acts as a potent disaggregase of chemically denatured and aggregated luciferase while yeast Hsp110 (Sse1) with yeast Hp70 (Ssa1) and Hsp40 (Ydj1) is less effective at disaggregating this same substrate (Rampelt et al., 2012).

There is, however, some controversy concerning the mechanism by which Hsp110 stimulates Hsp70/Hsp40 mediated disaggregation. A study by Shorter concluded that the substrate binding, ATP hydrolysis, NEF and Hsp70-binding activities of Hsp110 were all important for disaggregation, as mutations that abrogated any of these activities also abrogated Hsp110 stimulation of Hsp70/Hsp40 mediated disaggregation (Shorter, 2011). However, a subsequent study by Rampelt et al. challenged these conclusions by showing that mutations which reduced (human) or eliminated (yeast) Hsp110 ATPase activity had little to no effect on disaggregation. This study concluded that Hsp110 disaggregates proteins primarily or exclusively through its action as a NEF for Hsp70 (Rampelt et al., 2012). This controversy might potentially be resolvable by characterizing the effects of Hsp70 NEFs that are unrelated to Hsp110 and that lack the ATP hydrolysis or protein substrate binding activities displayed by Hsp70 family chaperones. However, the results of such experiments have also been contradictory: the Shorter study reported that NEFs such as Fes1 or Snl1ΔN could not stimulate Hsp70/Hsp40 mediated disaggregation, while the Rampelt study reported that Snl1ΔN and the NEF Bag1 could both stimulate Hsp70/Hsp40 mediated disaggregation, albeit to a lesser degree than Hsp110 and only with aggregates prepared under conditions that made them more amenable to chaperone-mediated disaggregation. The latter study also found that knockout of Hsp110, but not of Bag1, led to deficiencies in disaggregation and life-span reduction in heat-shocked nematodes (Rampelt et al., 2012), again suggesting that activities other than nucleotide exchange are important for the exceptional ability of Hsp110 in stimulating disaggregation. It is, however, possible that the differential disaggregating potencies of these NEFs do not reflect distinct activities in Hsp110, but quantitative differences in their nucleotide exchange action, as Hsp110 has been shown to have the most potent NEF activity (Raviol et al., 2006). The challenge to this interpretation is that no model has been developed that can quantitatively predict how varying levels of NEF activity, and the concomitant effects on the rate of different steps in the Hsp70 ATPase cycle, will affect Hsp70 refolding or disaggregation activity. Indeed, the Rampelt study titrated the amount of NEFs used in their reactions and showed that high concentrations of Hsp110 could inhibit disaggregation, likely by inducing a rate of ATP/ADP release from Hsp70 that was too fast for optimal activity. That the optimal Hsp110:Hsp70 ratio for disaggregation was found to be ~1:5 could itself be considered evidence that it is the NEF action of Hsp110 that is most important for disaggregation because this action is catalytic, while models in which the substrate binding or ATP hydrolyzing properties of Hsp110 are important are most easily envisioned to depend on formation of the 1:1 Hsp110:Hsp70 complex. Indeed, the formation of a such 1:1 complex in which the PBDs of each chaperone may bind a single substrate protein and/or transfer the substrate from one PBD to the other in a coordinated fashion has been proposed to underlie the exceptional refolding activity of 1:1 mixtures of mammalian Hsp110 and Hsp70 (Figure 2) (Mattoo et al., 2013).

However, if only the NEF action of Hsp110 is important for disaggregation then it seems that it should be possible to titrate NEFs of different potency–whether Hsp110, Bag1, or Snl1ΔN–to achieve the same NEF activity, and therefore disaggregation action, in any given reaction. The differences in the effectiveness of these NEFs in stimulating Hsp70/Hsp40 mediated disaggregation seem, nevertheless, to persist even when each is optimally titrated (Rampelt et al., 2012). This would suggest that some activity of Hsp110, other than its NEF action, is important for its exceptional disaggregating abilities, but what that activity may be and the quantitative contribution it makes to disaggregation may need further experimentation to be settled. However, the extant data do appear to agree on the following two points: (1) The combination of Hsp110, Hsp70, and Hsp40 acts an effective protein disaggregase (Shorter, 2011; Rampelt et al., 2012); (2) The mammalian Hsp110/Hsp70/Hsp40 combination is more effective at disaggregation than the yeast Hsp110/Hsp70/Hsp40 system (Rampelt et al., 2012). The latter observation is significant in light of the fact that fungi and plants contain an additional protein disaggregating chaperone (Hsp104/Hsp101; reviewed below) that is not found in animal cells. The protein disaggregating activity of the yeast Hsp104/Hsp70/Hsp40 combination is even more potent than that of the mammalian Hsp110/Hsp70/Hsp40 as the former can fragment and disaggregate the exceptionally stable protein aggregates known as prions, while the latter cannot (Shorter and Lindquist, 2006). Metazoans may therefore have evolved a more potent Hsp110-based disaggregation activity because they have lost Hsp104. Interestingly, it has recently been shown that yeast deficient for Hsp104, which is required for thermotolerance, are fitter than WT yeast when grown at 28°C (Escusa-Toret et al., 2013). From this it was concluded that the disaggregation activity of Hsp104 is so effective that it may sometimes dissociate productive protein complexes so that carrying it under non-stress conditions represents a significant burden. It was proposed that Hsp104 is maintained in these organisms because it is required for recovery of denatured/aggregated proteins that accumulate under the environmentally stressed conditions (most commonly, temperature extremes) that free-living fungi, as well as plants, frequently encounter. In contrast, animal cells encounter such environmental extremes to a lesser degree, and as a consequence may have dispensed with Hsp104 and, instead rely on an enhanced Hsp110 based disaggregation system.

### Hsp70 and clathrin coat dissociation

The apparently conflicting data on Hsp110 and Hsp70 mediated disaggregation may also reflect the challenge of reproducibly preparing denatured aggregates, as the nature and concentration of the chemical denaturant and the protein, and temperature and buffer conditions all strongly affect the size and secondary structure content of the aggregates and their susceptibility to disaggregation by chaperones (Diamant et al., 2000; Ben-Zvi and Goloubinoff, 2002; Lewandowska et al., 2007; Rampelt et al., 2012). It can therefore be useful to examine chaperone-mediated reactions that use naturally occurring homogeneous substrates rather than the heterogeneous aggregates prepared by chemical or thermal denaturation in the lab. One such reaction is Hsp70 mediated uncoating of clathrin coated vesicles. Such vesicles form as transient intermediates during endocytosis at plasma membranes or intracellular vesicular transport (Brodsky, 1988). The clathrin lattice (coat) that surrounds such vesicles is removed in an ATP dependent reaction by the constitutively expressed Hsc70 which is recruited to the coat by a J protein (auxilin or GAK) which, in addition to an Hsp70-binding J domain, contains a clathrin binding domain (Ungewickell, 1985; Ahle and Ungewickell, 1990; Greene and Eisenberg, 1990; Ungewickell et al., 1995, 1997; Holstein et al., 1996; Lee et al., 2006).

**Figure 3:**
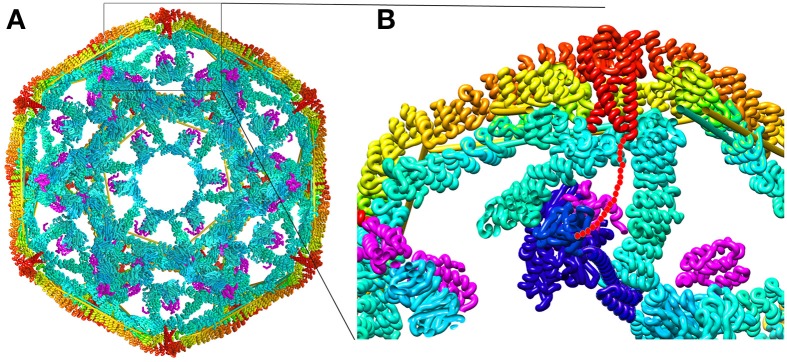
**Structural parameters of Hsp70 mediated clathrin coat disassembly. (A)** Cut-away view of the ~70 nm diameter clathrin coat with the J protein auxilin (magenta) bound [pdb 1XI5 (Xing et al., 2010)]. The coat is comprised of 36 triskelia, each of which contains 3 197 kD CHCs. The interior surface of the basket is cyan, with the exterior surface in yellow and orange. The C-termini of the CHCs in every triskelion associate to form a helical tripod (red) which is located under each vertex of the clathrin coat. **(B)** Expanded view of the boxed region from **(A)**. An Hsc70 [pbd 4B9Q (Kityk et al., 2012)] molecule (dark blue) modeled into the clathrin:auxilin coat on the basis of an Hsp70 NBD:auxilin J domain structure (Jiang et al., 2007) places its PBD in position to bind the flexible C-terminal tail (indicated by red dots) that emerges from the end of each tripod. This tail contains a single Hsp70 binding site required for Hsp70 mediated coat disassembly (Rapoport et al., 2008).

Structures of all the components (or close homologs) in this reaction are known, including the Auxilin J domain (Jiang et al., 2003), Hsp70*ATP (Kityk et al., 2012; Zhuravleva et al., 2012), Hsc70-Auxilin J-domain complex (Jiang et al., 2007), and the 700 Å diameter clathrin coat itself [alone (Fotin et al., 2004a) and with auxilin (Fotin et al., 2004b), or with auxilin and Hsc70 (Xing et al., 2010)], the latter obtained through a combination of cryoEM and X-ray crystallography. This allows modeling of snapshots in the reaction, particularly the step in which auxilin recruits Hsc70 to the basket (Figure 3). Such modeling positions the Hsc70 close to the C-terminus of the clathrin heavy chain (CHC) at the inner surface of the clathrin coat. The C-terminal tail of the CHC is flexible and contains a single Hsc70 binding motif (“QLMLT”) whose removal has been shown to abrogate the ability of Hsc70 to disassemble the coats (Rapoport et al., 2008). The kinetics of this reaction have been studied in ensemble experiments by light scattering (Scheurmann et al., 2008; Rothnie et al., 2011) and in single-particle experiments by fluorescence (Bocking et al., 2011), and have revealed that clathrin coat disassembly is fast, requiring only ~20 s at high Hsc70 concentrations. The number of Hsc70s needed to disassemble a coat can be considerably less than that required to saturate all the “QLMLT” binding sites present in the coat. The interactions between CHC molecules in the coat are highly ionic, and the stability of the coats can be modulated by pH, salt or mutation. It is found that more stable coats require binding of more Hsc70 molecules to induce disassembly, while less stable coats require fewer Hsc70s (Bocking et al., 2014). Another observation that is important in efforts to understand the disassembly mechanism is that auxilin or GAK, like most J proteins, bind to Hsc70 in its ATP state but, upon ADP hydrolysis, releases Hsc70 (Misselwitz et al., 1998; Liu et al., 2003).

These data allow us to develop the following description of the disassembly reaction. First, auxilin binds to clathrin coats and then recruits Hsc70*ATP. Upon ATP hydrolysis, Hsc70 releases its interaction with auxilin and is transferred to the flexible CHC C-terminal tail which is located at the each vertex of the clathrin coat, immediately adjacent to the inner surface of this structure. If buffer conditions favor coat disassembly then binding of an Hsc70 to one of every 3–6 of the CHC C-termini will induce coat disassembly; if conditions are coat stabilizing then Hsc70 recruitment persists until enough Hsc70 molecules are bound to induce disassembly. The force available to drive disassembly therefore appears to be a function of the number of Hsc70s that have bound to the coat (Bocking et al., 2014).

From the perspective of understanding the source of this force, in this and other reactions in which Hsp70s transduce chemical energy into mechanical transformation, the most salient elements may be the geometry of these set-ups. In the clathrin coat reaction this geometry is both distinct and strikingly similar to what we imagine it to be in a disaggregation reaction or protein translocation, the latter representing the other class of chemical energy to mechanical transformation reactions carried out by Hsp70s. The geometry is distinct inasmuch as in disaggregation reactions the Hsp70 has been assumed to bind on the outer surface of the aggregate and to pull proteins out the aggregate. In coat dissociation, the Hsp70 binds on the inner surface of the coat and may push, rather than pull, the coat apart. The geometry is similar since, in all these reactions, an Hsp70 appears to be recruited by a J protein to a flexible, extended peptide segment that emerges from structural wall (Liu et al., 2003). In the coat disassembly reaction this wall is the inner surface of the clathrin coat, and in the disaggregation or translocation reactions it is, respectively, the body of the aggregate or translocation pore. And, in all these reactions, once the Hsp70 clamps down on the extended peptide segment, which may be an unfolded protein loop or terminus, the J protein releases its interaction, leaving the Hsp70 effectively dangling from a flexible tether directly abutting a structural wall. How this geometry generates the force to induce coat disassembly, aggregate dissociation or protein translocation is not yet fully settled.

### Mechanisms of Hsp70 force generation

#### Brownian ratchet

**Figure 4:**
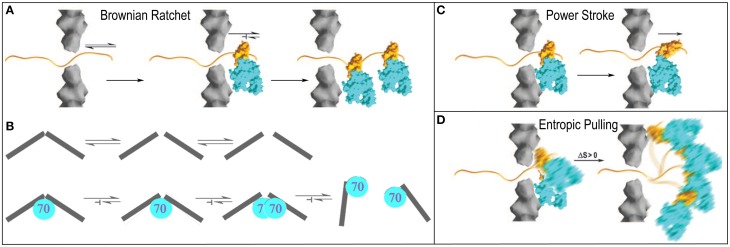
**Proposed mechanisms of Hsp70 mediated chemical to mechanical energy transformations (Sousa and Lafer, 2006). (A)** Brownian ratchet in the context of protein translocation. An unfolded protein can slide randomly back-and-forth through a translocation pore, but Hsp70s on one side can bind the protein so that it is trapped and eventually fully translocated to one side. **(B)** The ratchet in the context of clathrin coat disassembly. Fluctuations that loosen interactions in the coat occur spontaneously but when Hsp70 binds it blocks reversal of these fluctuations to the ground state. Accumulation of fluctuations causes disassembly (Xing et al., 2010). **(C)** Power-stroke. An Hsp70 binds to a protein emerging from a translocation pore and undergoes an ATP-hydrolysis coupled conformational change that pulls the protein through the pore. **(D)** Entropic pulling (De los Rios et al., 2006; Goloubinoff and De Los Rios, 2007). Binding of an Hsp70 to a polypeptide segment emerging from a translocation pore restricts the freedom of motion of the Hsp70. Movement of the Hsp70 away from the pore leads to greater freedom of motion and entropy, and to a favorable free energy change. Mechanisms are presented in the context of protein translocation but are easily extended to reactions in which Hsp70 pulls proteins out of aggregates.

The Brownian or thermal ratchet mechanism postulates that Hsp70 does not actively induce or drive directional mechanical transformation in its substrates. Instead, it merely asymmetrically captures fluctuations that occur spontaneously. This mechanism is easiest to apprehend in the context of protein translocation where an unfolded protein may slide randomly back and forth in a translocation pore, but where Hsp70 molecules are only recruited to one side of the pore. Whenever random sliding exposes an Hsp70 binding site on the side of the pore that contains Hsp70 molecules, it may be bound by an Hsp70 which, because of its size, sterically prevents the protein from sliding back up the pore (Matlack et al., 1999). Cycles of such passive sliding and Hsp70 binding eventually result in translocation of the entire protein to the Hsp70 side of pore (Figure 4A). This is the mechanism which has been advanced to explain clathrin coat disassembly, where it is proposed that Hsc70 binds against the interior surface of the coat and sterically blocks reversal of spontaneous fluctuations in the coat that loosen the interactions that hold the CHC molecules together (Xing et al., 2010). Trapping of enough of these loosening fluctuations, perhaps accompanied by binding of additional Hsc70 molecules as loosening of the coat makes room, ultimately leads to coat disassembly. Crucially, the presence of the Hsc70 molecules is not proposed to amplify either the amplitude or frequency of these fluctuations. As in the protein translocation mechanism, it is the asymmetric capturing of spontaneous fluctuations that gives direction to the mechanical transformation of the substrate (Figure 4B).

#### Power-stroke

In contrast, a power-stroke mechanism envisions that an Hsp70 binds its substrate and then, at some point in the ATP hydrolysis reaction, undergoes a conformational change that pulls on the substrate to effect mechanical transformation (Sousa and Lafer, 2006). Though this mechanism cannot be fully ruled out, it is problematic in that it requires that the Hsp70 bind or rest against a structure that acts as a fulcrum against which the Hsp70 can push, but data indicate (Liu et al., 2003) that Hsp70 is recruited to its substrates via a mechanism that leaves it dangling on a flexible tether with no apparent fulcrum against which it might push (Figure 4C).

#### Entropic pulling

Brownian ratchet and power stroke mechanisms typically define the two extremes proposed to explain macromolecular mediated chemical-to-mechanical energy transformations. A few years ago, this debate was interrupted by the proposal of a mechanism that has been dubbed “entropic pulling,” but which is equally well described as an excluded volume effect (De los Rios et al., 2006; Goloubinoff and De Los Rios, 2007). The entropic pulling model points out that when Hsp70 binds to a flexible polypeptide element that abuts a structural wall, its freedom of motion is restricted. Movement of the Hsp70 away from the wall, which results in pulling on the bound polypeptide, is associated with a favorable entropy increase, and a free energy decrease that is proportional to the 1st derivative of the entropy change as a function of distance from the wall (Figure 4D). The potential force generated by this effect was estimated to be as great at 10–20 piconewtons (De los Rios et al., 2006), which compares favorably to the 5–10 piconewtons generated by ATP-hydrolyzing motor proteins like kinesin or myosin (Svoboda and Block, 1994). Entropic pulling can therefore generate directional pulling forces as large as, or larger than, those generated by active power stroke mechanisms, but without the requirement for the complex conformational change coupling required in those mechanisms.

However, while the entropic pulling model has a number of attractive features, it is extremely challenging to definitively establish or disprove mechanisms in motor protein action. Until resolved by further experimentation, all three proposed models for Hsp70 force generation remain viable.

## The Hsp104/ClpB disaggregase

### The AAA+ family of proteins

The Hsp104/ClpB chaperones are members of the Clp/Hsp100 family of AAA+ (ATPases associated with various cellular activities) proteins. AAA+ proteins are part of a vast superfamily of P-loop NTPases identified by the presence of a 200–250 residue core NTPase domain characterized by the presence of a P-loop; Walker A and B helical nucleotide binding elements; sensor motif 1 and arginine finger motifs (Iyer et al., 2004; Mogk et al., 2004). Within this superfamily, the AAA+ proteins are identified by having a helical C-terminal extension (the C-domain) which contains sensor motif 2 which is characterized by the presence of well-conserved arginine or lysine (Neuwald et al., 1999; Ammelburg et al., 2006). AAA+ proteins couple the energy of NTP hydrolysis to conformational (or associational state) changes in proteins, nucleic acids and even lipid membranes. They may therefore be considered the major NTP hydrolysis driven, conformational change inducing engines of the cell (Hanson and Whiteheart, 2005).

Hsp104/ClpB chaperones (which also include mitochondrial Hsp78 and plant Hsp101) contain two NBDs, which makes them members of the class I group of the Clp/Hsp100 family (Doyle and Wickner, 2009). The two nucleotide binding domains are separated by a coiled coil middle (M) domain and preceded by an N-terminal (N) domain. Other members of this class include ClpA and ClpC, which differ structurally from the Hsp104/ClpB chaperones in that they lack (ClpA), or have much smaller (ClpC), M-domains. Like the single NTPase-domain class II Clp/Hsp100 proteins ClpX and HspIU, ClpA and ClpC use ATP hydrolysis to drive unfolding of proteins which they then feed to the proteases with which they associate.

### Models for the mechanism of Hsp104/ClpB action

#### Polypeptide translocation through a central pore

**Figure 5:**
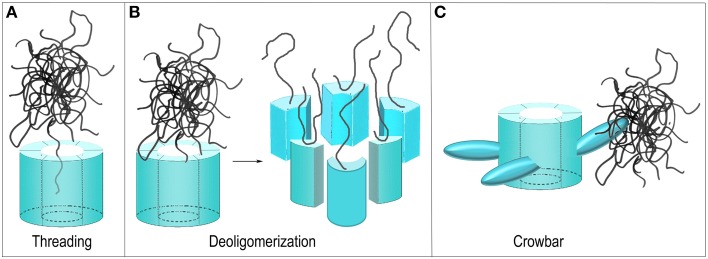
**Proposed mechanisms of ClpB/Hsp104 mediated protein disaggregation. (A)** Threading: The ClpB/Hsp104 hexamer engages a protein in an aggregate and pulls it through its central pore to separate it from the aggregate. **(B)** Deoligomerization: A ClpB/Hsp104 hexamer binds an aggregate and then deoligomerizes so that each ClpB/Hsp104 protomer pulls a single bound protein away from the aggregate. **(C)** Crowbar: The mobile M-domains of the ClpB/Hsp104 hexamer engage and break up the aggregate.

Though the Hsp104/ClpB chaperones do not associate with proteases, they show mechanistic similarities to the Clp proteins which do. Hsp104/ClpB form hexameric rings arranged around a central pore large enough to allow extended polypeptides, but not folded protein domains, to pass. Via a mechanism that is not yet fully understood, they use the energy of ATP hydrolysis to extract a protein from an aggregate and progressively unfold and translocate it through the central pore (Figure 5A). This mechanism received support from an experiment showing that, when ClpB was genetically fused to the ClpP ring protease, the resulting fusion acted as a disaggregating/unfolding proteolytic machine, indicating that ClpB was able feed the unfolded proteins into the ClpP proteolytic chamber (Weibezahn et al., 2004). This aspect of the of the ClpB/Hsp104 mechanism is therefore reminiscent of that of another group of hexameric AAA+ proteins—the nucleic acid helicases—which similarly use the energy of ATP hydrolysis to unwind nucleic acid secondary structure by translocating single stranded DNA or RNA through their central pores (Hanson and Whiteheart, 2005).

#### Aggregate dissociation by Hsp104/ClpB deoligomerization

Not all AAA+ proteins work by translocating their substrates through a central pore and alternative models for the Hsp104/ClpB mechanism have been considered. The GTPase Dynamin, for example, is related to the AAA+ proteins and its function is to sever the membrane neck that connects a nascent vesicle to the plasma membrane during endocytosis. It does so by oligomerizing to form a ring around this neck and subsequently constricting to sever the neck and release the vesicle (Pawlowski, 2010; Chappie et al., 2011). One proposed model for Hsp104/ClpB action can be considered a reversal of the Dynamin mechanism. Hsp104/ClpB monomers could oligomerize around a protein aggregate but then subsequently deoligomerize, with each chaperone monomer pulling an associated substrate protein with it to break up the aggregate (Figure 5B) (Werbeck et al., 2008). This mechanism was effectively ruled out by an experiment in which it was shown that an Hsp104 hexamer with disulfides engineered to link the monomers together could still dissociate protein aggregates (Biter et al., 2012), demonstrating that deoligomerization was not required for Hsp104 action.

#### Aggregate dissociation by M-domain “crowbars”

Yet another model proposed that the M-domains—which are flexible and essential for disaggregation, and which project out from the side of the ClpB/Hsp104 hexamer—act as “crowbars” that engage and break up the aggregate (Figure 5C) (Glover and Tkach, 2001). However, to introduce markers for the M-domains for EM studies, the Tsai group genetically introduced T4 lysozyme molecules into the Hsp104 M-domains. Unexpectedly, this fusion protein was active in protein disaggregation, a result that argued that the M-domain was not acting as a crowbar in this process since its fusion to the bulky T4 lysozyme molecule would be expected to disrupt such a function (Lee et al., 2010). Even more surprisingly, while efficient protein disaggregation by ClpB/Hsp104 requires the presence of Hsp70 and the Hsp40 J protein, it was found that M-domain fusion with T4 lysozyme activated the protein disaggregation activity of Hsp104 so that Hsp70/Hsp40 were no longer required for efficient disaggregation.

### Regulation of ClpB/Hsp104 action

#### The M-domains regulate ClpB/Hsp104 activity

**Figure 6:**
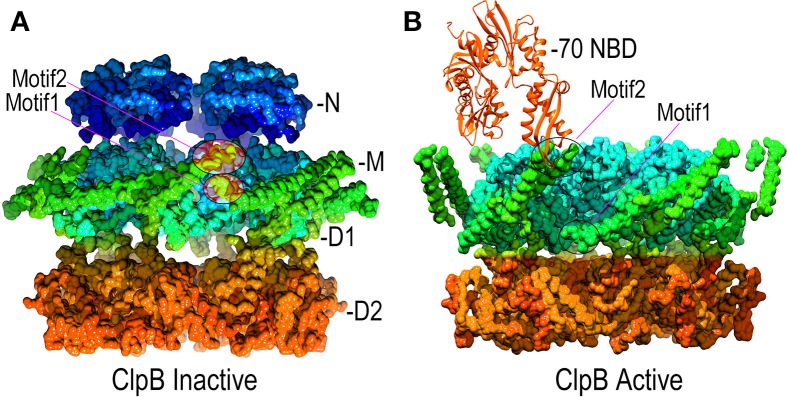
**Mechanisms of M-domain and Hsp70 regulation of ClpB/Hsp104 activity. (A)** Inactive ClpB hexamer conformation [pdb 4D2Q (Carroni et al., 2014)]. Interactions between motif 1 and motif 2 at, respectively, the base and tip of the helical M-domains (green) hold the M-domains together as a continuous band of protein around the D1 ring. **(B)** Active ClpB hexamer conformation [pdb 4D2X (Carroni et al., 2014)]. M-domains tilt away from each other and motif1:motif2 interactions are disrupted. Hsp70 (orange) binds motif2 and may activate ClpB/Hsp104 by disrupting motif1:motif2 interactions.

Point mutations in the M-domains have been identified which, like the T4 lysozyme fusion, activate ClpB/Hsp104 disaggregase activity, and M-domain mutations that inhibit ClpB/Hsp104 have also been identified (Oguchi et al., 2012; Lee et al., 2013; Rosenzweig et al., 2013; Carroni et al., 2014). These results indicate that the M-domains regulate ClpB/Hsp104 action and recent EM studies of ClpB have revealed how such regulation may work (Carroni et al., 2014). The M-domains form extended coiled-coil structures, and in ClpB hexamers bearing M-domain mutants that lock ClpB in an inactive state, the M-domains lie with their long axes perpendicular to the central pore axis so that the head of one M-domain can interact with the tail of another (Figure 6A). In this configuration, the M-domains effectively wrap the hexamer with a continuous ribbon of protein which may restrict the conformational changes needed to activate the disaggregase activity. In contrast, mutations that activate ClpB cause the M-domains to tilt, thereby breaking M-domain:M-domain interactions and increasing M-domain mobility (Figure 6B). This movement of the M-domains may relieve repression of ClpB activity, possibly by relaxing the constraint on conformational changes and motion in the other domains of the hexamer.

#### Hsp70s activate Hsp104/ClpB by binding to the M-domain

Hsp70/Hsp40 itself has protein remodeling activity, so it's possible that this activity is responsible for the enhanced disaggregation activity when Hsp70/Hsp40 are added to reactions with Hsp104/ClpB. For example, Hsp70/Hsp40 may act on aggregates to partially break them up or free proteins from the aggregate, which are then bound by ClpB/Hsp104 and actively threaded through the latter's central pore (Zietkiewicz et al., 2006; Acebron et al., 2009). Alternatively, Hsp70/Hsp40 could act subsequent, rather than prior, to ClpB/Hsp104, and could bind the unfolded protein as it emerges from the ClpB/Hsp104 pore to prevent its re-aggregation and allow it to fold properly (Goloubinoff et al., 1999). These models are not mutually exclusive and both Hsp70/Hsp40 mediated remodeling of aggregates and inhibition of re-aggregation of proteins newly freed from aggregates may make important contributions to protein disaggregation by ClpB/Hsp104.

However, the observation that efficient disaggregation by bacterial ClpB requires bacterial Hsp70 (DnaK) and Hsp40 (DnaJ), while fungal Hsp104 requires fungal Hsp70/Hsp40, indicated that efficient disaggregation also requires specific interactions between ClpB/Hsp104 and their co-specific Hsp70/Hsp40 partners (Sielaff and Tsai, 2010; Winkler et al., 2012; Lee et al., 2013). The observation that fusion of T4 lysozyme to the M-domain activated Hsp104 disaggregation activity suggested that the M-domain is the target through which Hsp70 activates Hsp104 (Lee et al., 2010). This was borne out by identification of mutants in the M-domain that abrogate interaction with Hsp70 (Oguchi et al., 2012), by showing that swapping the ClpB and Hsp104 M-domains swapped their Hsp70 specificities, and by experiments showing that binding of StrepTactin protein to a strep tag introduced into the M-domain activated Hsp104 disaggregase activity, just as was seen with the T4 lysozyme fusion (Sielaff and Tsai, 2010; Lee et al., 2013). EM studies have recently illuminated how the M-domains regulate ClpB activity and have also indicated how Hsp70 participates in this regulation: the head-to-tail interactions that lock M-domains together in a repressive configuration involve motif 1 (at base or tail of one M-domain) interactions with motif 2 (at the tip or head of an adjacent M-domain). The Hsp70 nucleotide binding domain (NBD) binds to motif 2 of the M-domain. Hsp70:M-domain interactions and M-domain:M-domain head-to-tail contacts are therefore mutually exclusive. In the absence of Hsp70, ClpB is in equilibrium between active and repressed states, with the repressed state being favored. Binding of Hsp70 to the active ClpB conformation blocks it reversion to the repressed state, and shifts ClpB into the active conformation (Figure 6B) (Seyffer et al., 2012; Rosenzweig et al., 2013; Carroni et al., 2014).

Though contributions from Hsp70/Hsp40 intrinsic protein remodeling activity to protein disaggregation are likely, the primary mechanism by which Hs70/Hsp40 enhance ClpB/Hsp104 action is now believed to be through Hsp70 binding to the M-domains (Acebron et al., 2009; Winkler et al., 2012). Importantly, since this binding involves the Hsp70 NBD, the Hsp70 PBD remains available for protein substrate binding even when Hsp70 is bound to ClpB/Hsp104. It is easy to imagine that this allows Hsp70 to bind a protein aggregate and subsequently recruit Hsp104 so that the latter's disaggregation function is specifically activated only when it is presented proximal to a protein aggregate.

### Mechanism of polypeptide translocation through the ClpB/Hsp104 pore

**Figure 7:**
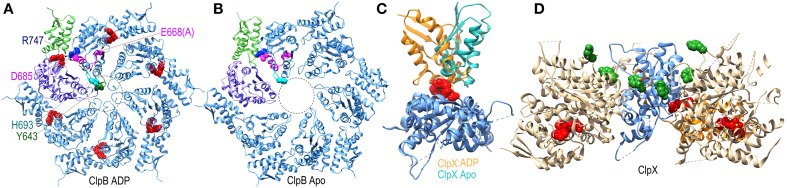
**Nucleotide dependent conformational changes underlying protein threading through the ClpB/Hsp104 pore. (A)** A ClpB D2 hexamer ring assembled from the structure of ClpB D2 with ADP (red) bound [pdb 4FCV (Biter et al., 2012)]. The small and large subdomains of one of the D2 domains are colored green and purple, respectively, to illustrate how nucleotide binds at the interface of these two subdomains. ADP bound to this domain is sensed by the arginine finger (R747 in blue) of the adjacent domain. R747 contacts D685 of the ISS motif (magenta). Also in magenta are the proximal D9 helix and E668 (mutated to ala in this structure) of the Walker B motif which contacts bound ADP. These residues sit at the base of a β-hairpin which contains H693 at its tip, and which is packed against the pore loop with its conserved tyrosine (Y643 in green). The pore loops lie within the area defined by the dotted circle and are ordered in the ADP-bound structure. **(B)** Apo ClpB D2 hexamer ring [pdb 4FCT (Biter et al., 2012)]. The pore loops are disordered and not visible in the apo state. **(C)** Superimposed structures of apo and ADP-bound ClpX [pdb 3HWS (Glynn et al., 2009)] protomers show how ADP binding induces large rotations between the large and small subdomains. **(D)** Structure of an asymmetric ClpX hexamer illustrates how the ADP induced changes in the orientation between large and small subdomains shift the positions of the protomers and of the substrate-binding pore loops (highlighted by the green space-filling renderings of the conserved tyrosines at the tips of the loops) in the hexamer (the large and small subdomains of one of the protomers are colored, respectively, blue and orange).

The ATPase domains of AAA+ proteins contain loops which line the interior of the ring pore (Figure 7A), and are characterized by a conserved ϕ-Xxx-Gly motif at their tips (in the ClpB/Hsp104 chaperones the conserved hydrophobe is a tyrosine). The pore loops bind polypeptide substrates, and the interactions and conformation of a pore loop are regulated by the nucleotide state of both its own (cis) domain and the adjacent (trans) domain (Lum et al., 2004; Schlieker et al., 2004; Biter et al., 2012). Central to sensing the nucleotide state of the trans domain is the arginine finger residue of the cis domain, which inserts into the ATP binding site of the trans domain to contact the bound ATP. ATP interactions with this arginine shift its side-chain and alter its interaction with a conserved aspartate in the cis domain. This aspartate is part of the intersubunit signaling (ISS) motif and its interactions with the arginine finger may change the position of the ISS motif residues, as well as the immediately N-terminal D9 helix and Walker B motif whose conserved glutamate binds nucleotide in the cis domain. The ISS motif and D9 helix are at the base of a β-hairpin which packs against and buttresses the pore loop. Thus, two paths, one from the arginine finger and one from the Walker B motif, are proposed to converge on the ISS motif and D9 helix to relay information on the nucleotide state of, respectively, the trans and cis domains in the hexameric ring (Figure 7A). Evidence for nucleotide effects on pore loop conformation is that in a structure of nucleotide-free ClpB the pore loops are disordered (Figure 7B), while in an ADP-bound structure the loops become ordered and visible (Biter et al., 2012).

More global changes in ATPase domain conformation due to nucleotide binding are seen in a ClpX hexamer structure (Glynn et al., 2009). Though composed of 6 chemically identical protomers, this hexamer is conformationally asymmetric, with ADP bound in only 4 of the ClpX molecules. Each protomer is conformationally distinct, but the largest differences are between the ADP-bound and apo protein conformations which differ primarily in the relative orientation of the large and small subdomains of each protomer due to the binding of ADP at the interface of these two subdomains (Figure 7C). As a consequence of these largely rigid-body rotations, the packing of each protomer with its partners in the hexamer is altered and their relative positions shifted resulting, in turn, in shifts in the positions of the pore loops relative to the pore loop axis (Figure 7D). ClpX is a protease and member of the single-ring (class II) AAA+ family, rather than the double-ring (class I) family of which ClpB/Hsp104 are members, so the degree to which ClpB/Hsp104 may undergo similar inter-subdomain rotations is unclear, but nucleotide binds similarly at the interface of the large and small subdomains in the disaggregases (Figure 7A) so such rotations seem likely, and the occurrence of conformational asymmetry during the reaction cycles of AAA+ proteins is consistent with the observation of asymmetric and sub-stoichiometric nucleotide binding to the hexamers in solution (Hersch et al., 2005).

Polypeptide translocation would therefore be envisioned to involve sequential rounds of ATP hydrolysis in which a peptide segment would first be bound by the pore loop(s) of a domain that has bound ATP, since ClpB/Hsp104 displays the highest affinity for substrates when in the ATP state. ATP hydrolysis would trigger translocation coupled to pore loop movement, and ADP release would cause the cis pore loop to release the polypeptide, which would then be bound by the pore loop of the trans domain in the ATP state and the cycle would repeat. The degree to which the mechanism would involve direct stepwise transfer of the polypeptide from one pore loop to another (Biter et al., 2012) vs. a mechanism in which the loops would associate more loosely with the polypeptide and move it through the pore by waves of rowing-like motions (Doyle et al., 2013) is unclear, and may differ in different AAA+ proteins.

### Impact of protein aggregate structure on ClpB/Hsp104 function

#### ClpB/Hsp104 are uniquely able to dissociate secondary-structure rich aggregates

Not all protein aggregates are equally susceptible to chaperone-mediated dissociation. Aggregates of denatured luciferase that are differentially enriched in β-sheet structures can be prepared by using different concentrations of GuHCl in the denaturation reaction. Amorphous aggregates with little secondary structure can be dissociated by the combination of an Hsp70, Hsp40 and a NEF, while aggregates with high β-sheet content require the presence of ClpB as well (Lewandowska et al., 2007). Aggregate size, as opposed to secondary structure content, does not appear to be important in determining the requirement for ClpB. It has also been shown that the related ClpX unfolds proteins much more effectively when it can engage the protein at a terminus than when it binds the protein at an internal peptide segment, leading to the conclusion that ClpX unfolds proteins, in a process that typically burns ~1 ATP per unfolded residue, by progressive disruption of secondary structure rather than by global unfolding (Kenniston et al., 2004).

This progressive mechanism may explain why ClpB/Hsp104 can disassemble extremely stable (SDS resistant) protein aggregates, but cannot unfold stably folded protein domains. This has been shown in experiments in which a labile, denatured protein is fused to a more stable, natively-folded protein domain. In such a case ClpB/Hsp104 will unfold and disaggregate the labile, denatured portion of the fusion but not the more stably folded protein (Haslberger et al., 2008). Presumably, ClpB/Hsp104 can progressively unfold the denatured part of the fusion even when local regions of secondary structure are encountered, but when it reaches the stable, natively-folded protein its further progress is blocked, as this would require a more global disruption of the protein's tertiary structure. It is not, however, the case that the inability to unfold stable, native proteins is an absolute limitation of the AAA+ chaperone mechanism. The AAA+ ClpC, which is closely related to ClpB but normally collaborates with the ClpP protease (Turgay et al., 1997, 1998), can fully unfold both the labile as well as the stable portions of the fusions that block ClpB progress (Haslberger et al., 2008). The inability of ClpB/Hsp104 to unfold stable, native domains may therefore be a mechanism for discriminating between well-folded native domains and less stable, non-native structures.

#### Hsp104 in prion biology

The conclusion that ClpB/Hsp104 is able to unfold secondary structure, especially β-sheet structure, would be consistent with the role of Hsp104 in prion propagation. Prions represent a particular type of ordered protein aggregate that is rich in amyloid (indefinitely extended intermolecular β-sheet) structure and capable of self-propagation via a mechanism in which prion fragments act as seeds onto which soluble prion proteins can coalesce to grow the insoluble prion (Colby and Prusiner, 2011). They are most familiar as the causative agents of human diseases like Kuru or Creutzfeld-Jacob (mad cow) disease (Aguzzi et al., 2008). However, in some organisms transitions between soluble (non-prion) and insoluble prion forms of an endogenous protein are part of the normal biology of those organisms (Lindquist, 1996; Si et al., 2003; Halfmann and Lindquist, 2010). Such prions have been most extensively studied in yeast and include the transcriptional regulator Ure2 and the translation termination factor Sup35 and Rnq1, whose prion forms are designated, respectively, [URE3], [PSI+], and [PIN+] (Patino et al., 1996; Tuite and Lindquist, 1996). Propagation of Ure2 and Sup35 prions in yeast is dependent on normal levels of endogenous Hsp104, but this dependency is complex because overexpression of Hsp104 causes loss of Sup35, but not Ure2, prions (Shorter and Lindquist, 2006).

The role of Hsp104 in propagating prions is attributed to both its protein binding/unfolding and translocation/disaggregation activities. *De novo* formation of prions from soluble Ure2 or Sup35 can occur spontaneously, but is strongly accelerated *in vitro* by Hsp104 (Shorter and Lindquist, 2004). Surprisingly, the ability of Hsp104 to stimulate *de novo* prion formation *in vitro* requires ATP binding, but not ATP hydrolysis. The mechanism remains obscure, but it may be that Hsp104 can bind and hold soluble Ure2 or Sup35 in a partially unfolded state which can subsequently oligomerize into species that can proceed to prion formation. In addition to stimulating *de novo* prion formation, Hsp104 accelerates prion growth via a mechanism that requires ATP hydrolysis. Hsp104 does this by fragmenting prions, probably by pulling single protomers out of a prion fibril so that the fibril is split and two fresh surfaces are exposed that can then template the growth of more insoluble prion (Shorter and Lindquist, 2008; Doyle et al., 2013). The ability of Hsp104 to break up prions is remarkable given that these aggregates are stable enough to resist solubilization by ionic detergents like SDS.

Surprisingly, unlike what is observed with most protein aggregates, the disaggregating effect of Hsp104 on prions *in vitro* is not stimulated by Hsp70 and/or Hsp40 (Shorter and Lindquist, 2006). The basis for this distinction is unclear, but it has been shown that Hsp104 preferentially binds lys/arg rich peptides and Sup35 contains a lys-rich element which is exposed on the surface of the Sup35 prion fibril. It is therefore possible that Hsp104 binding to these lys-rich sequences is sufficient to both recruit Hsp104 to the fibril and to activate its disaggregating activity in the absence of Hsp70 (Shorter and Lindquist, 2004). However, the Hsp70-independent effect of Hsp104 on prions *in vitro* is at odds with data showing that the ability of Hsp104 to propagate Sup35 prions in live cells requires the yeast Hsp40 homolog, Sis1 (Tipton et al., 2008). Experiments have also shown that recruitment of Hsp104 to both non-prion (luciferase) and prion (Rnq1/[PIN+]) aggregates *in vivo* requires the yeast Hsp70 homolog, Ssa1, which is first recruited to these aggregates by an Hsp40 (Sis1) and subsequently recruits Hsp104 (Winkler et al., 2012). Hsp104 mediated luciferase disaggregation and prion fragmentation were both similarly dependent on Sis1 and Ssa1. Interestingly, binding of Hsp104 to Sup35 ([PSI+]) prions *in vivo* did not require Ssa1. This Ssa1-independent binding involved the Hsp104 N domain and may depend on Hsp104 N domain's intrinsic affinity for the lys-rich segment of Sup35 mentioned previously. However, this binding was non-productive: in the absence of Ssa1, Hsp104 associates stably with Sup35 prions but does not fragment them. The reasons for the differences in *in vivo* vs. *in vitro* Hsp70/Hsp40 requirements for Hsp104 mediated prion binding and fragmentation remain unclear, but the data would indicate that the *in vivo* pathways follow the canonical mechanism of chaperone coordination in which an Hsp40 J protein first recruits and activates an Hsp70, which subsequently recruits and activates an Hsp104. That this pathway can be short circuited *in vitro* need not be considered a challenge to our understanding of the normal physiological pathways of this coordination.

The requirement for Hsp104 to sustain prion propagation *in vivo* is interpreted in terms of a need to fragment prions so as to generate seeds to sustain prion multiplication in the face of their dilution by cell growth and division (Shorter and Lindquist, 2008). The observation that overexpression of Hsp104 can eliminate (cure) Sup35 ([PSI+]) prions from yeast could be most simply attributed to the effects of an overly vigorous fragmentation activity: a certain amount of Hsp104 activity is required to fragment prions to generate fresh seed surfaces and sustain prion growth, but too much can lead to complete prion solubilization and loss of prion propagation. However, this does not explain why Hsp104 overexpression causes loss of [PSI+] but not [URE3] prions. *In vitro* and *in vivo* experiments both argue against the prion resolubilization mechanism as the basis for Hsp104 overexpression mediated curing of [PSI+]. *In vitro*, high levels of Hsp104 fragment Ure2 prions into small fibrils that retain the ability to seed new prion growth, while Sup35 prions are fragmented into soluble protein and amyloid-like aggregates that cannot seed such growth (Shorter and Lindquist, 2006). However, *in vivo* overexpression of Hsp104 is observed to result in excess binding of Hsp104 to Sup35 prions and displacement of Ssa1 binding. Since binding of Hsp104 without Ssa1 is non-productive for prion fragmentation this results in loss of [PSI+] since new prion seed fragments are not generated (Winkler et al., 2012). Hsp104 overexpression does not similarly cure [URE3] because Hsp104 does not engage in non-productive (Ssa1-independent) binding to Ure2 prions. Thus, the *in vitro* and *in vivo* data explain the ability of Hsp104 overexpression to cure [PSI+], but not [URE3], by different mechanisms: *in vitro*, Hsp104 is active but fragments Sup35 prions into non-seeding species while fragmenting Ure2 prions into seeding-competent species; while *in vivo*, Hsp104 binds non-productively to Sup35 prions and outcompetes Ssa1 but is unable to bind Ure2 prions without Ssa1. The basis for this difference is unclear.

### NSF mediated SNARE complex dissociation

#### NSF dissociates SNARE complexes

ClpB/Hsp104 homologs are found in bacteria, fungi and plants but, with the exception of the mitochondrial compartment, not in metazoans (Doyle et al., 2013). However, all eukaryotes express a protein called NSF (N-ethylmaleimide-sensitive factor), an AAA+ family member which, like ClpB/Hsp104, is hexameric and displays an N-domain and 2 nucleotide binding domains (D1 and D2), but, unlike ClpB/Hsp104, has no M-domain (Zhao et al., 2010). A primary function of NSF is dissociation of the SNARE complexes which drive membrane fusion (Otto et al., 1997; Sudhof and Rothman, 2009). SNAREs are membrane proteins with predominately helical cytoplasmic domains (Sutton et al., 1998). Helical elements of a SNARE protein in one membrane can associate with helices from a SNARE protein in another to form coiled-coils which bring the two membranes into close apposition so that they can fuse (Sudhof and Rothman, 2009). The resultant intermolecular interactions are extensive and SNARE complexes are very stable: like prions they are resistant to SDS solubilization.

**Figure 8:**
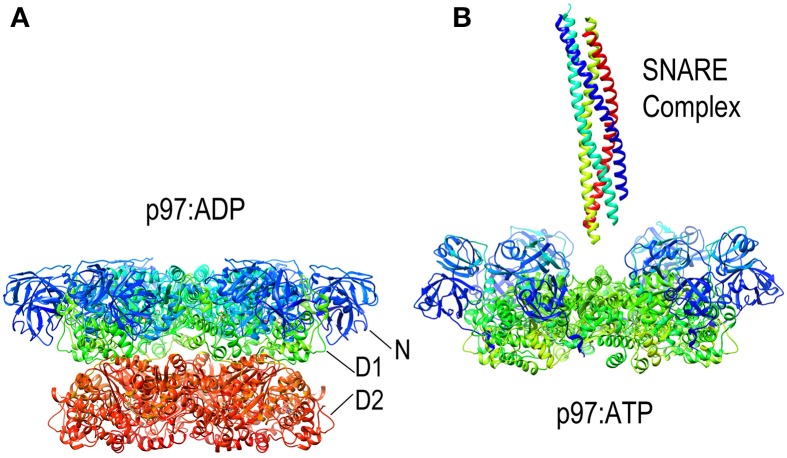
**Nucleotide dependent conformational changes in p97/NSF. (A)** Structure of the p97 hexamer in its ADP state [pdb HU2 (Tang et al., 2010)]. **(B)** Structure of the p97 hexamer [missing the D2 ring; pdb 3CF3 (Davies et al., 2008)] shows how the N-domains (blue and cyan) move toward the central pore in the ATP state. A SNARE complex (pdb 1URQ) is shown in the orientation in which it binds NSF; p97 does not itself disassemble SNARE complexes but is shown here as illustrative of this class of AAA+ proteins as atomic resolution structures of NSF hexamers have not been determined, but EM studies have shown NSF to undergo similar nucleotide dependent conformational changes.

Since it lacks an M-domain, it is unsurprising that NSF action is neither activated by Hsp70 nor uses that chaperone to bind its substrates. Instead, NSF binds SNARE complexes primarily via interactions between the NSF N-domain and the αSNAP (soluble NSF attachment protein), which binds to the surface of the SNARE complexes (Chang et al., 2012). Binding of ATP to NSF induces the N-domains to move from the periphery of the D1 ring of NSF toward the pore at the center of the NSF hexamer (as is also seen in the NSF homolog p97; Figure 8). It is in the ATP state that the N-domains are appropriately positioned and competent to bind αSNAP. SNARE complexes are long coiled coil structures that bind with their N-termini in the center of NSF and with their long axes parallel to the axis that runs through the NSF central pore. A cryo-EM model of an NSF-αSNAP-SNARE (20S) complex suggests that 3 αSNAP molecules bind around the SNARE complex and contact the N-domains of the NSF (Chang et al., 2012).

#### Mechanisms of NSF mediated SNARE complex dissociation

**Figure 9:**
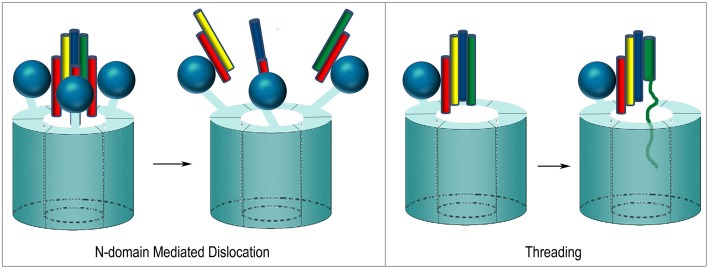
**Proposed mechanisms of NSF mediated SNARE complex dissociation**. In N-domain mediated dislocation, three N-domains bind 3 αSNAP molecules on the outside of the SNARE complex and, upon ATP hydrolysis, the N-domains move and pull apart the SNARE complex. In the threading mechanism, only one αSNAP is productively bound to the SNARE complex and recruits the complex to the NSF hexamer via the N-domain, which then engages a SNARE complex protomer and progressively unfolds and threads it through the central pore until the complex dissociates.

At least two mechanisms have been proposed for how NSF disassembles the SNARE complex. One is based on the observation that, upon ATP hydrolysis, the N-domains move outwards and away from the center of NSF as the D1 and D2 rings rotate relative to each other (Davies et al., 2008; Chang et al., 2012; Tang and Xia, 2013). The outwards movement of the N-domains is proposed to pull on the αSNAP molecules which, in turn, pull the protomers of the SNARE complex apart and away from each other (Figure 9). In contrast to this “global untwisting” model, a processive threading mechanism, similar to that of ClpB/Hsp104, has been proposed on the basis of biochemical experiments characterizing the kinetics, affinities, and stoichiometries of NSF-mediated disassembly reactions with a variety of WT and engineered SNARE complexes (Figure 9) (Cipriano et al., 2013; Vivona et al., 2013). These studies indicate that the functional stoichiometry of αSNAP:SNARE complex binding is 1:1 (only one αSNAP is actually involved in recruiting NSF to the SNARE complex), and that the 3:1 ratio observed by EM reflects binding of additional αSNAP molecules to the 6-fold symmetric NSF without any direct contact with the SNARE complex, which lacks any 3-fold or higher order symmetry. Once αSNAP recruits NSF to the SNARE complex, pore lining loops in the D1 domain, including a loop that contains a conserved YXG motif and is homologous to the substrate binding loop of ClpB/Hsp104, are proposed to engage the extended N-terminus of one of the SNARE complex protomers and unfold and thread it through the central pore in a processive reaction that hydrolyzes ~1 ATP for every residue that is unfolded and translocated. ATP hydrolysis is coordinated with substrate binding because interactions with αSNAP and the SNARE proteins activate ATPase activity 20–30-fold (Vivona et al., 2013). SNARE complexes can contain two (binary) or three (ternary) SNARE proteins but it is expected that threading of one protein either completely or partially through NSF would be sufficient to disrupt the complex. The two models of NSF action are not mutually exclusive: it is possible that N-domain motions coupled to ATP hydrolysis exert a force on αSNAP(s) which serves to loosen the SNARE complex enough to allow the D1 ring to engage one of the protomers and initiate its processive unfolding (alternatively, the ATP coupled movements of the N-domains may be important simply to position them for SNARE complex binding).

### Relating AAA+ mediated protein disaggregation, complex dissociation and translocation

#### A role for AAA+ proteins in protein translocation

Like Hsp70s, which participate not only in protein complex or aggregate dissociation but also in protein translocation between cellular compartments, AAA+ proteins are also involved in moving proteins. A homolog of NSF, the AAA+ p97 (also called valosin-containing protein or VCP in mammals, and Cdc48 in yeast) extracts ubiquitinated, misfolded proteins from the ER and translationally stalled proteins from the ribosome, and transfers them to the proteasome for degradation (Wang et al., 2004; Brandman et al., 2012). Recruitment of p97 to its ER associated substrates involves its N-domain, which binds the Ufd1-Npl14 cofactor (in other p97-mediated reactions, other co-factors such as p47 similarly bind to the p97 N-domain). Binding of ubiquitinated substrates to the p97-Ufd1-Npl14 complex depends on direct interactions with both p97 and its co-factor and activates ATP hydrolysis by p97 (Ye et al., 2003). An arginine rich pore lining loop in the D2 ring of p97 is involved in substrate binding and may be important for substrate unfolding, while a loop bearing conservative aromatic residues at its tip is proposed to bind unfolded hydrophobic substrates and to be functionally analogous to the structurally similar pore loop of ClpB/Hsp104 (DeLaBarre et al., 2006). However, p97 mechanism may be distinct from NSF in that the p97 D1 ring appears to play primarily a structural role, and it is the D2 ring which accounts for p97 ATP hydrolysis, and engages and translocates protein substrates (DeLaBarre et al., 2006), while in NSF the opposite is true: the D1 ring is the mechanically active, ATP-hydrolyzing element while interprotomer interactions in the D2 ring are important for stabilizing the hexamer (Zhao et al., 2010). The absence of hydrolytic activity or nucleotide dependent conformational changes in the p97 D1 ring, and the general insensitivity of p97 function to D1 mutations has led to the conclusion that p97 does not completely thread substrates through its central pore as NSF and ClpB/Hsp104 are proposed to do. Instead, it appears that the D2 ring loops engage the substrate and then either thread it only through the D2 ring and allow it to emerge through spaces between the D1 and D2 rings (“partial threading”), or substrates may simply be bound in the D2 pore before being transferred to the proteasome (“surface binding”) (DeLaBarre et al., 2006; Buchberger, 2013). However, the archaeal p97 homolog VAT is able to fully unfold globular proteins, and both its D1 and D2 ring have ATP hydrolysis activity, with the D1 ring displaying a pore loop with a conserved tryptophan that is crucial for substrate binding and protein unfolding. This has led to the conclusion that, unlike eukaryotic p97, archaeal p97 functions by threading proteins through its central pore, as do NSF and ClpB/Hsp104 (Gerega et al., 2005).

#### Common features of ClpB/Hsp104 disaggregase and NSF/p97 complex disassembly and translocation mechanisms

While many details of the AAA+ chaperone mechanisms have yet to be elucidated, the elements common to the mechanisms of the general disaggregases (ClpB/Hsp104) and the specific complex remodelers (NSF/p97) are extensive and define the general features of how these chaperones work. First, they all display N-domains that are involved in substrate binding, either directly or through adaptor proteins. N-domain interactions are especially important for NSF/p97 function, and while ClpB/Hsp104 N-domains are dispensable for disaggregation and prion propagation, the N-domain of Hsp104 is involved in binding to the Sup35 prion. The lesser importance of N-domain:substrate interactions for ClpB/Hsp104 vs. NSF/p97 may reflect the unique presence in the former of the mobile M-domains which bind to Hsp70 to recruit ClpB/Hsp104 to its substrates. The binding of Hsp70 not only recruits, but stimulates ClpB/Hsp104 activity. NSF and p97 ATPase activity is also stimulated upon protein substrate binding, and all these data highlight the tight regulation and coordination of action in these chaperones which insures that they are activated primarily when bound to an appropriate substrate in an appropriate context. Affinity for substrates is highest when the chaperone is ATP-bound and lower or not detectable in the ADP/Apo state. ATP binding and hydrolysis each result in changes in both the overall conformation of the hexamer (movements of the N- and M-domains, rotations of the D1 and D2 rings relative to each other), and in the conserved loops that line the interior of the central pore. These loops engage the substrate protein and their movement is believed to represent the power-stroke that pulls proteins through the pore. Conformational changes in the loops are regulated both by the nucleotide state of the ATPase domain in which they occur (the cis domain) and by the adjacent (trans) domain in the same ring, which allows for the coordinated transfer of the substrate protein from one domain to the next in a processive stepping or wavelike rowing mechanism that is coupled to ATP hydrolysis and moves the protein either partially (p97) or fully (NSF and ClpB/Hsp104) through the chaperone. The role of interactions between the D1 and D2 rings (as opposed to between domains within each ring) in AAA+ chaperone mechanisms are less well understood and may differ between chaperones.

This mechanism is effective in disassembling protein:protein associations that are extremely stable as demonstrated by the SDS insolubility of prions and SNARE complexes, and the compactness and extent of intermolecular interactions in these intermolecular β-sheet and coiled coil structures. However, this does not necessarily imply that this mechanism generates exceptionally large forces. Instead, it may be that it is the geometry of force application which renders this mechanism so effective. Rather than dissociate a protein:protein interaction by global disruption of multiple, extensive bonding contacts, the threading mechanism of these AAA+ chaperones processively and incrementally disrupts small-scale, local interactions until a point is reached where the entire complex dissociates. In such a mechanism, each ATP hydrolysis event is therefore responsible for disruption of only a limited number of non-covalent interactions as has been quantitatively measured in NSF mediated SNARE complex disassembly or ClpX mediated protein unfolding which each consume, on average, 1 ATP for every amino residue that is unfolded and threaded through the chaperone.

### A small non-ATP hydrolyzing disaggregase

That dissociation of protein aggregates and complexes would require ATP-hydrolyzing macromolecular machines is unsurprising since we expect these to be mechanical, energy-dependent processes. However, small non-ATP hydrolyzing chaperones that can dissociate protein aggregates have been identified. The most well understood of these is the 38-kDa subunit of the chloroplast signal recognition particle (cpSRP43), which disaggregates the light-harvesting chlorophyll a/b-binding proteins (LHCPs) (Jaru-Ampornpan et al., 2010). LHCPs are some of the most abundant proteins on earth and are highly hydrophobic with 3 trans-membrane helices in each protomer. The nuclear-encoded LHCPs are translated in the cytoplasm and cpSRP43 is part of the machinery that subsequently delivers them to the chloroplast thylakoid membranes. It would be expected that the hydrophobic LHCPs would depend on chaperones to inhibit their aggregation during transport through an aqueous milieu before they reach their homes in the membrane.

**Figure 10:**
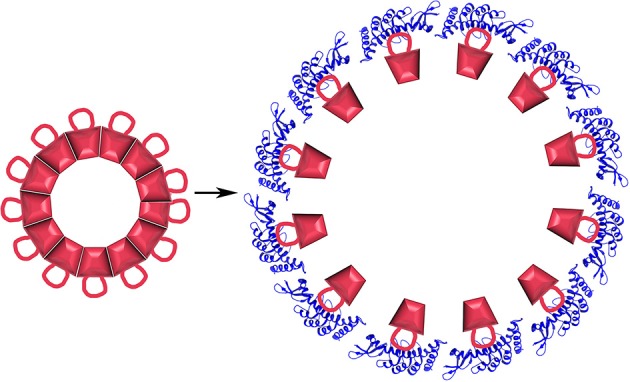
**Protein disaggregation by a non-ATP hydrolyzing chaperone**. LHCP (red) aggregates into discoid structures with a conserved 18 residue loop displayed on its surface. Binding of cpSRP43 [blue; pdb 3DEO (Stengel et al., 2008)] causes aggregate dissociation.

What is less expected is that the cpSRP43 chaperone can not only inhibit aggregation but can also dissociate LHCP aggregates. This dissociation has been shown to depend on a sequence specific interaction between a hydrophobic groove (centered on Y204) of cpSRP43 and a conserved 18-residue loop (L18: residues 152–169) situated between the 2nd and 3rd TM helices of LHCP. In addition to this sequence specific interaction, non-sequence specific interactions between the LHCP TM helices and hydrophobic regions of cpSRP43 also contribute to cpSRP43:LHCP binding (Jaru-Ampornpan et al., 2013). Dissociation of LHCP aggregates by cpSRP43 depends not only on these interactions, but also on the structure of the LHCP aggregates themselves. These are neither fully amorphous, nor as highly structured as amyloid filaments. Instead, LHCP aggregates into discoid structures 10–20 nm in diameter in which the TM segments are largely buried while the more polar L18 loop is exposed on the surface of the aggregate (Nguyen et al., 2013). Though highly stable (resistant to 2% SDS), the surface exposure of the L18 loop allows binding of multiple cpSRP43 proteins to the aggregate which, in a cooperative reaction driven solely by the binding energy of the cpSRP43:LHCP interaction, drive dissociation of the aggregate into 1:1 cpSRP43:LHCP complexes (Figure 10).

It should be remarked that the physiological significance of this dissociation reaction has yet to be established. A newly translated LHCP may normally associate to form a transit complex with a cpSRP43:cpSRP54 heterodimer without proceeding through an aggregated state. However, the defined nature of the LHCP aggregate, which presents the L18 loop on its surface, suggests a mechanism evolved to allow rescue of these off-pathway aggregates by interaction with a specific chaperone.

## Summary

Molecular chaperones can unfold and disaggregate proteins, dissociate specific protein complexes and pull proteins through translocation pores. A small ATP-independent chaperone that can dissociate a specific aggregate may be the exception to the rule that these reactions are driven by ATP hydrolysis (though such ATP-independent disaggregases may be more widespread than is currently realized). Hsp70/Hsp110 and ClpB/Hsp104 represent the two major classes of ATP-dependent disaggregating chaperones.

ClpB/Hsp104 are members of the AAA+ family of proteins which assemble into hexameric rings and translocate extended polypeptides or nucleic acids through their central pores in order to dissociate protein aggregates and complexes, and unwind nucleic acid secondary structure. They do this by coupling nucleotide binding and hydrolysis to protein domain rotations and conformational changes in substrate binding pore loops. This is a true power-stroke mechanism as the movement of a pore loop and its bound substrate along the pore axis is directly coupled to a step in the ATP hydrolysis cycle. Threading of the substrate through the central pore appears to be common to reactions in which ClpB/Hsp104 dissociates amorphous aggregates as well as β-sheet rich prions, and in reactions in which the related NSF protein dissociates SNARE complexes, though p97 may also operate via a mechanism in which the substrate is only partially threaded or bound to the pore of its D2 ring. However, even in this case pore loops that are structurally similar to those in ClpB/Hsp104 are proposed to engage substrate and undergo nucleotide-dependent conformational changes to dislocate ubiquitinated substrates from the ER or stalled ribosomes to allow their transfer to the proteasome.

However, the mechanism of initial substrate recognition and binding differs in these enzymes. NSF and p97 utilize their mobile N domains to bind substrates via an interaction with an intermediary (adaptor) protein. Though the N-domain of Hsp104 can also recruit Hsp104 to the Sup35 prion, the N-domains of the disaggregases are likely less important for substrate recruitment. Instead ClpB/Hsp104 appear to rely on Hsp70/Hsp40 to recruit substrates. This may reflect the fact that ClpB/Hsp104 operate on heterogeneous aggregates composed of different proteins which may be more readily recognized by general Hsp40/Hp70 chaperone functions, while NSF or p97 work with a limited set of substrates that display specific recognition motifs. Regulation presents another distinction as ClpB/Hsp104 activity is repressed by their M domains, which are unique to the ClpB/Hsp104 family. These extended coiled-coil domains establish head-to-tail intermolecular interactions to form a ring of protein that encircles the ClpB/Hsp104 hexamer. Hsp70 binding to the M domain competitively displaces this interaction, mobilizes the M domains and activates the disaggregase. The recruitment function of Hsp70/Hsp40 is obligatory *in vivo* but is dispensable *in vitro* if ClpB/Hsp104 are activated by M domain mutations that disrupt the head-to-tail interactions, but the significance of this difference is not understood.

Hsp70, together with its Hsp40 J protein and NEF co-chaperones, has a modest disaggregase activity that is markedly amplified by Hsp110, a divergent Hsp70 family member with distinct substrate binding properties that also acts as a NEF for Hsp70. The structural mechanism by which Hsp70 binds and releases its substrates is now well understood, but how Hsp70 and Hsp110 cooperate in disaggregation, and the roles of each chaperone in the process, are not resolved and are areas of active investigation. Also still in question is the exact molecular kinetic mechanism by which Hsp70s generate the forces that move or structurally alter their substrates. Brownian mechanisms in which Hsp70s asymmetrically capture spontaneous fluctuations in a substrate, and power-strokes in which an ATP dependent conformational change in the chaperone directly drives movement or structural change in a bound substrate have been proposed. A third mechanism (entropic pulling) that harnesses excluded volume effects for these reactions is compelling, but remains speculative and it is unclear how it might operate in the context of clathrin coat disassembly, where Hsp70 binds on the inside of the coat and pushing might be more effective than pulling to induce coat disassembly.

Geometry appears to be a critical element in these mechanisms. J proteins recruit Hsp70s to their substrates by mechanisms that leave the Hsp70 bound to a flexible polypeptide segment immediately abutting a structural wall. This geometry can generate an entropic pulling force though, in the context of clathrin coat disassembly, it has also been proposed that it could block reversal of loosening fluctuations in the clathrin coat. For the ClpB/Hsp104 disaggregases, engagement of a substrate loop or terminus and threading provide a mechanism for progressive and incremental disruption of local interactions which allows these enzymes to dissociate extremely stable, secondary-structure rich structures like prions, which might be impossible to disrupt globally and cannot be dissociated by the Hsp70/Hsp110 system. This alerts us to another recurrent theme in disaggregase function: the need to control these enzymes and only activate them in the appropriate context. For the Hsp70s this is done primarily by having the J protein recruit substrate to the Hsp70 which is then synergistically activated by interactions with both the J protein and the substrate. Thus, only a substrate with features attractive to both the J protein and the Hsp70 will be acted upon with maximal activity. ClpB/Hsp104, at least *in vivo*, similarly harnesses this double-selection system as ClpB/Hsp104 is itself recruited and activated by Hsp40/Hsp70, and then likely adds a third layer of selection since the substrate must display a free terminus or flexible loop that can be engaged by the disaggregase. That such regulation is critical is highlighted by the observation that organisms that can limit the environmental extremes to which their cells are exposed have dispensed with ClpB/Hsp104, and that carrying Hsp104 is a significant burden for yeast growing at constant temperature in a laboratory. It seems that these potent disaggregases need to be controlled to insure that they do not disrupt *appropriate* interactions.

### Conflict of interest statement

The author declares that the research was conducted in the absence of any commercial or financial relationships that could be construed as a potential conflict of interest.
